# *Trichinella spiralis* Paramyosin Binds to C8 and C9 and Protects the Tissue-Dwelling Nematode from Being Attacked by Host Complement

**DOI:** 10.1371/journal.pntd.0001225

**Published:** 2011-07-05

**Authors:** Zhifei Zhang, Jing Yang, Junfei Wei, Yaping Yang, Xiaoqin Chen, Xi Zhao, Yuan Gu, Shijuan Cui, Xinping Zhu

**Affiliations:** Department of Parasitology, School of Basic Medical Sciences, Capital Medical University, Beijing, People's Republic of China; Instituto Butantan, Brazil

## Abstract

**Background:**

Paramyosin is a thick myofibrillar protein found exclusively in invertebrates. Evidence suggested that paramyosin from helminths serves not only as a structural protein but also as an immunomodulatory agent. We previously reported that recombinant *Trichinella spiralis* paramyosin (*Ts*-Pmy) elicited a partial protective immunity in mice. In this study, the ability of *Ts*-Pmy to bind host complement components and protect against host complement attack was investigated.

**Methods and Findings:**

In this study, the transcriptional and protein expression levels of *Ts*-Pmy were determined in *T. spiralis* newborn larva (NBL), muscle larva (ML) and adult worm developmental stages by RT-PCR and western blot analysis. Expression of *Ts*-Pmy at the outer membrane was observed in NBL and adult worms using immunogold electron microscopy and immunofluorescence staining. Functional analysis revealed that recombinant *Ts*-Pmy(r*Ts*-Pmy) strongly bound to complement components C8 and C9 and inhibited the polymerization of C9 during the formation of the membrane attack complex (MAC). r*Ts*-Pmy also inhibited the lysis of rabbit erythrocytes (E_R_) elicited by an alternative pathway-activated complement from guinea pig serum. Inhibition of native *Ts*-Pmy on the surface of NBL with a specific antiserum reduced larvae viability when under the attack of complement *in vitro*. *In vivo* passive transfer of anti-*Ts*-Pmy antiserum and complement-treated larvae into mice also significantly reduced the number of larvae that developed to ML.

**Conclusion:**

These studies suggest that the outer membrane form of *T. spiralis* paramyosin plays an important role in the evasion of the host complement attack.

## Author Summary

Trichinellosis is a serious food borne parasitic disease caused by the consumption of meat contaminated with the infective larvae of *Trichinella spiralis.* The ability of the tissue-dwelling parasite to evade the host complement attack is essential for its survival and for establishing infection in the host. This study describes the expression of paramyosin, a muscular protein in invertebrates, on the surface of *Trichinella spiralis* and its role in the defense against the host complement attack as a survival strategy. Using a specific antiserum, expression of *Trichinella spiralis* paramyosin was detected on the outer membrane of the adult worms and newborn larvae. Functional analysis revealed that recombinant *Trichinella spiralis* paramyosin protein strongly bound human complement components C8 and C9 and inhibited the formation of the complement membrane attack complex. Neutralization with a specific antiserum greatly impaired the protective effect of paramyosin on the viability and infectivity of *Trichinella spiralis* newborn larva when under attack by complement. These studies suggest that the outer membrane form of *Trichinella spiralis* paramyosin plays an important role in the evasion of the host complement attack and is therefore a good target for vaccine and pharmaceutical development.

## Introduction

Trichinellosis is one of the common parasitic zoonoses and is a serious public threat in both developing and developed countries [1]-[6]. *Trichinella spiralis* (*T. spiralis*) infection is initiated by the consumption of meat contaminated with infective muscle larvae (ML). With the aid of host gastric juice, ML are released from cysts and migrate to the small intestine where they develop into adult worms in 2–3 days. Five days post-infection, gravid females begin to produce newborn larvae (NBL), which penetrate the intestinal mucosa and enter the lymphatic vessels and bloodstream [7]. NBL travel through capillaries to various organs and finally invade the muscles, where they form cysts. During the life cycle of *T. spiralis* in the host, all developmental stages are exposed to host complement, which is the first line of defense against pathogenic organisms and is a functional bridge between the innate and adaptive immune responses [8].

The ability to evade complement attack is essential for the survival of parasites within their respective hosts [9]. As early as 1911, the presence of complement-fixing antigens from larvae of *T. spiralis* was reported in antiformin extracts of pepsin-digested rat muscle [10]. Complement -fixing antigens have since been used to diagnosis of trichinosis of trichinellosis [11], [12]. Subsequent studies have reported that the complement elements C3, C5 [13], C1q, C8 and C9 [14], [15] directly bind the ML of *T. spiralis.* All three stages of *T. spiralis* are capable of activating complement via the classical or alternative pathways [14], or the lectin pathway [16]. However, it is still unknown whether the activation of the complement is detrimental or beneficial to the parasite. NBL might be the most potent activators [13]. Molecules or structures on the outermost cuticle/epicuticle of the parasite directly bind complement and appear to protect the parasite from an attack by inhibiting the formation of the membrane attack complex (MAC) [14], [15]. Rats with normal levels of C6 or those with a C6-deficiency have similar susceptibilities to infection by *T. spiralis*. However, C3, C8 and C9 were found to bind worms, suggesting that *T. spiralis* has efficient mechanisms for protecting against complement attack [15]. However, the precise molecular basis for this resistance is still unknown.

Paramyosin is a thick myofibrillar protein found exclusively in invertebrates [17]. Experimental evidence has shown that paramyosin from helminths serves not only as a structural protein but also as an immunomodulatory agent [18]–[22]. It has been reported that paramyosin from *Taenia solium* inhibits C1 function [18]. Paramyosin from *Schistosoma mansoni* acts as an immunological defense molecule by binding C1q [18], the Fc fragment of IgG [19], C8 and C9 [20]–[21]. Recently, paramyosin from *Clonorchis sinensis* was shown to bind both human collagen and C9 [22]. In our previous study, a full-length cDNA encoding *T. spiralis* paramyosin (*Ts*-Pmy) was cloned by immunoscreening an adult *T. spiralis* cDNA library with infected immune sera [23], Recombinant *Ts*-Pmy (r*Ts*-Pmy) elicited partial protective immunity against a *T. spiralis* larval challenge in BALB/c mice [24]. In the present study, we investigated ability of r*Ts*-Pmy to bind to host complement components and to protect against host complement attack. Our data show that r*Ts*-Pmy binds complement components C8 and C9 and inhibits the complement-mediated killing of NBL, providing more evidence that *Ts*-Pmy plays an important role in the evasion of the host immune response to facilitate the survival of *T. spiralis* in its host.

## Materials and Methods

### Animals

All experimental animals were purchased from Laboratory Animal Services Center of Capital Medical University (Beijing, China). All experimental procedures were reviewed and approved by the Capital Medical University Animal Care and Use Committee and were consistent with the NIH Guidelines for the Care and Use of Laboratory Animals.

### Parasites and antigen preparation

*T. spiralis* (ISS 533 strain) was maintained in female ICR mice. ML were recovered from the muscles of infected mice by a standard pepsin/hydrochloric acid digestion method as described previously [14]. Adult worms were obtained from the intestine of a rat infected orally with 800 *T. spiralis* ML [25]. NBL were obtained from fertile female adult worms cultured overnight in RPMI 1640 at 37°C. Crude somatic extracts of the different stages of *T. spiralis* were prepared by conventional methods [26], and the protein concentration was determined by the BCA assay (Pierce,USA).

### RT-PCR analysis

Total RNA was extracted from *T. spiralis* ML, adult worms and NBL with an RNAeasy mini kit (Qiagen, Germany) according to the manufacturer's instructions. Total first-strand cDNAs were reverse transcribed from the total mRNAs using a Sensiscript Reverse Transcription kit (Qiagen, Germany). The specific forward primer (5′- ACC AAC TGA GGG CTT TGC A-3′) and reverse primer (5-′ AAT ATT CAT GTC CTT CTT CCA TCA C-3′), based on *Ts*-Pmy coding sequence of 1830–2730 bp, were used to amplify *Ts-pmy* cDNA fragments (900 bp) from reverse transcribed total cDNA from different developmental stages of *T. spiralis* using a PCR kit (TaKaRa, China). Reactions without the addition of reverse transcriptase were used as negative controls. The amplified products were analyzed in 2% agarose gels and stained with DNA Green (Tandz, USA).

### Expression of recombinant paramyosin (r*Ts*-Pmy) and preparation of the anti-r*Ts-*Pmy antibody

r*Ts*-Pmy was expressed in *E. coli* BL-21(DE3) using the pET-28a expression system (Novagen, USA) and purified with Ni-affinity chromatography (Qiagen, USA), as described previously [23]. The antiserum against r*Ts*-pmy was raised in rabbits immunized three times with 150 µg r*Ts*-Pmy. The monoclonal antibody against r*Ts*-Pmy (mAb 7E2) was obtained using a conventional hybridoma technique, and IgG was purified with HiTrap rProtein A affinity columns (Amersham Biosciences, USA; data not shown).

### Western blot analysis

Crude somatic extracts of *T. spiralis* ML, adult worms and NBL were subjected to SDS-PAGE on a 12% acrylamide gel and transferred onto a PVDF membrane (Millipore, USA). After being blocked with a 5% (w/v) skim milk solution in Tris-buffered saline (TBS) containing 0.05% Tween-20 (Sigma, USA) (TBS-T) for 1 hour at room temperature, the membrane was incubated with mAb 7E2 at a concentration of 50–100 ng/mL in TBS-T. Peroxidase-conjugated goat anti-mouse IgG (1∶5,000; Sigma, USA) was used as secondary antibody. The reaction was visualized with enhanced chemiluminescence reagent (Pierce, USA) and exposed to a BioMax film (Kodak, USA). In some experiments, the Odyssey two color infrared imaging system was used according to manufacturer's instructions.

### Immunolocalization

#### Immuno-gold labeling and electron microscopy

To examine whether *Ts*-Pmy was located on the surface of the parasite, the *T. spiralis* adult worms and NBL were fixed with 5% paraformaldehyde and 0.1% glutaraldehyde in phosphate buffered saline (PBS) for 72 hours at 4°, rinsed four times for 20 min each in PBS and immuno-labeled with mAb 7E2 (1 µg/mL in PBS) and gold-conjugated protein A (15 nm; Zymed Laboratories Inc., USA). Normal mouse serum at the same dilution was used as the control. The immuno-labeled worms were postfixed in 1% osmium tetroxide for 1 hour and dehydrated in a series of alcohol and acetone before being embedded in Araldite and Epon. Ultrathin sections were cut and stained with uranyl acetate and lead citrate, and examined using a JEM 1011 transmission electron microscope (TEM) at 80 kV.

#### Immunofluorescence labeling

To determine if *Ts*-Pmy is located on the surface of intact worms, whole adult worms and NBL of *T. spiralis* were incubated with rabbit anti-r*Ts*-Pmy antiserum or normal rabbit serum for 3 hours at 37°C. After being washed three times with RPMI 1640, the worms were treated with fluorescein isothiocyanate-conjugated goat anti-rabbit IgG (Sigma) (1∶100 in RPMI 1640) for 1 hour. The worms were washed three times with RPMI 1640 and examined under a fluorescence microscope (Leica, Germany).

### Complement-mediated killing of NBL *in vitro*

A total of 150 NBL released by adult female worms in culture were pretreated with heat-inactivated (56° for 30 min) rabbit anti-r*Ts-*Pmy serum, the same serum pre-absorbed with r*Ts*-Pmy or heat-inactivated normal rabbit serum (NRS) (2, 20 or 40 µL) in a total volume of 50 µL in a 96-well plate for 1 hour at room temperature. Heat-inactivated rabbit antiserum against *Ts*87 was used as a non-relevant antibody control (40 µL). Then, 100 µL of freshly pooled normal guinea pig serum (NGS) was added as a complement source into each well, and the incubation was continued for 24 hours in a 5% CO_2_ incubator at 37°C. Heat-inactivated NGS (INGS) was added as a control. NBL mortality was monitored under an inverted microscope based on motility (The worms without any movement during 30 seconds of observation and total stretch-out were scored as dead) and the fluorescent staining of the DNA-binding dye SYTOX Green (Invitrogen, USA) [27]. Experiments were run in triplicate. Percent mortality was calculated as described elsewhere [21], [28].

### Passive transfer experiments of NBL

To determine the viability of NBL treated with anti-r*Ts-*Pmy serum and NGS, treated NBL were passively transferred into BALB/c mice intravenously. Briefly, 2000 NBL per group were pretreated with 100 µL of heat-inactivated anti-r*Ts*-Pmy rabbit serum, the same serum pre-absorbed with r*Ts*-Pmy or heat-inactivated NRS, and incubated with 400 µL of fresh NGS for 12 hours in a 5% CO_2_ incubator at 37°C. INGS were used as a control. After being incubated with NGS or INGS, NBL were washed with serum-free RPMI-1640, re-suspended in 0.25 ml of PBS and injected into the lateral tail vein of BALB/c mice (8 mice for each group) as described elsewhere [29]. Muscle larvae burdens were collected and counted on the 26th day after injection [14].

### Hemolytic assay of the alternative complement pathway

Complement-mediated lysis of rabbit erythrocytes (E_R_) was performed via the alternative complement pathway as described by Hong et al. [14]. To test whether *Ts*-Pmy acts as an inhibitor or neutralizer of the complement activated by the alternative pathway, fresh NGS (6%) were pre-incubated with various amounts of r*Ts*-Pmy (0, 10, 20 or 40 µg) in Mg-EGTA solution (5 mM MgCl_2_, 10 mM EGTA) for 30 min before adding the mixture to fresh, washed E_R_ (3×10^8^) in 0.1 mL of GVB (Veronal-buffered saline, pH 7.4, containing 0.1% gelatin and 0.02% NaN_3_) for 30 min at 37°C. Lysis was stopped by adding 1 mL of cold GVB containing 10 mM EDTA. After being centrifuged at 4,400×*g* for 10 min at 4°C, the amount of hemoglobin released into the supernatant was measured at 412 nm, and the percent lysis relative to the number of cells lysed completely by water was calculated.

### r*Ts*-Pmy binding to C8 and C9

To determine whether r*Ts*-Pmy binds to C8 and C9,purified human C8,C9 (Merck,Germany) and non-relevant control BSA (Sigma,USA) (1 µg each) were subjected to SDS-PAGE under reducing conditions and transferred to a PVDF membrane. After blocking with 5% milk in TBS-T, the membrane was incubated with 10 mL of r*Ts*-Pmy (5 µg/mL in TBS-T) for 3 hours at 37°C and then with mAb 7E2 (50 ng/mL) for 1 hour at room temperature. Peroxidase-conjugated goat anti-mouse IgG (1∶4,000; Sigma,USA) was used as the secondary antibody. Bands were visualized with ECL (Pierce,USA). To determine the specific binding of *Ts*-Pmy to C8 and C9, r*Ts*-87 was used as a control to react with C8 and C9 that had been transferred to a PVDF membrane and probed with anti-*Ts*87 antiserum. For the reciprocal experiment, the same amount of r*Ts*-Pmy and BSA (1 µg ) was transferred to a PVDF membrane and incubated with C9 (0.5 µg/mL) for 2 hours at 37°C. After being washed with TBS-T, the membrane was incubated with monoclonal anti-C9 antibody (1∶4,000; Abcam, USA). To determine the competition between the soluble C9 and blotted C9 for binding to *Ts*-Pmy, r*Ts*-Pmy was pre-incubated with C9 1∶6 (w/w, 5 µg/mL of r*Ts*-Pmy and 30 µg/mL of C9) for 1 hour at 37°C before being incubated with the membrane. The membrane was then reacted with mAb 7E2 (50 ng/mL) and IRDye-800CW-conjugated goat anti-mouse IgG (1∶10,000; LI-COR, Germany).

### C9 polymerization assays

To determine the effect of r*Ts*-Pmy on Zn^2+^-activated C9 polymerization [30], 3 µg of C9 was pre-incubated with various amounts of r*Ts*-Pmy at 37°C for 40 min and incubated with 50 µM ZnCl_2_ in 20 mM Tris buffer, pH 7.2 for 2 hours at 37°C. Inhibition of C9 polymerization was shown by SDS-PAGE on a 2.5 to 25% acrylamide gradient gel under reducing conditions; the gel was visualized by staining with Coomassie blue [31] or analyzed by western blot with an anti-C9 antibody. A non-relevant *T. spiralis* antigen *Ts*87 was used as a control.

r*Ts*-Pmy inhibition of C9 polymerization on E_R_ was performed as reported by Tschopp et al. [32]. One hundred microliters of normal human serum (NHS) supplemented with 5 µg of C9 was pre-incubated with 30 µg of r*Ts*-Pmy for 40 min at 37°C prior to the addition of 3×10^6^ E_R_ and continued incubation for 1 hour at 37°C. Lysed cells were washed three times with 3 mL of TBS containing 5 mM EDTA (pH 8.0) and centrifuged for 20 min at 4,800×*g* at 4°C. Sediments were washed three times with 3 mL of 0.5 mM PBS (pH 8.0). Lysed cell pellets were analyzed by SDS-PAGE under reducing conditions on a 2.5 to 15% gradient acrylamide gel followed by either Coomassie blue staining or Western blot with an anti-C9 antibody.

### Statistical analysis

Results were expressed as the mean ± SD. Differences between groups were assessed by SPSS 10.0 (SPSS Inc., USA) using One-Way ANOVA; *p*<0.05 was considered to be statistically significant.

## Results

### 1. Expression and localization of *Ts*-Pmy in different developmental stages of *T*. *spiralis*

**Figure 1:**
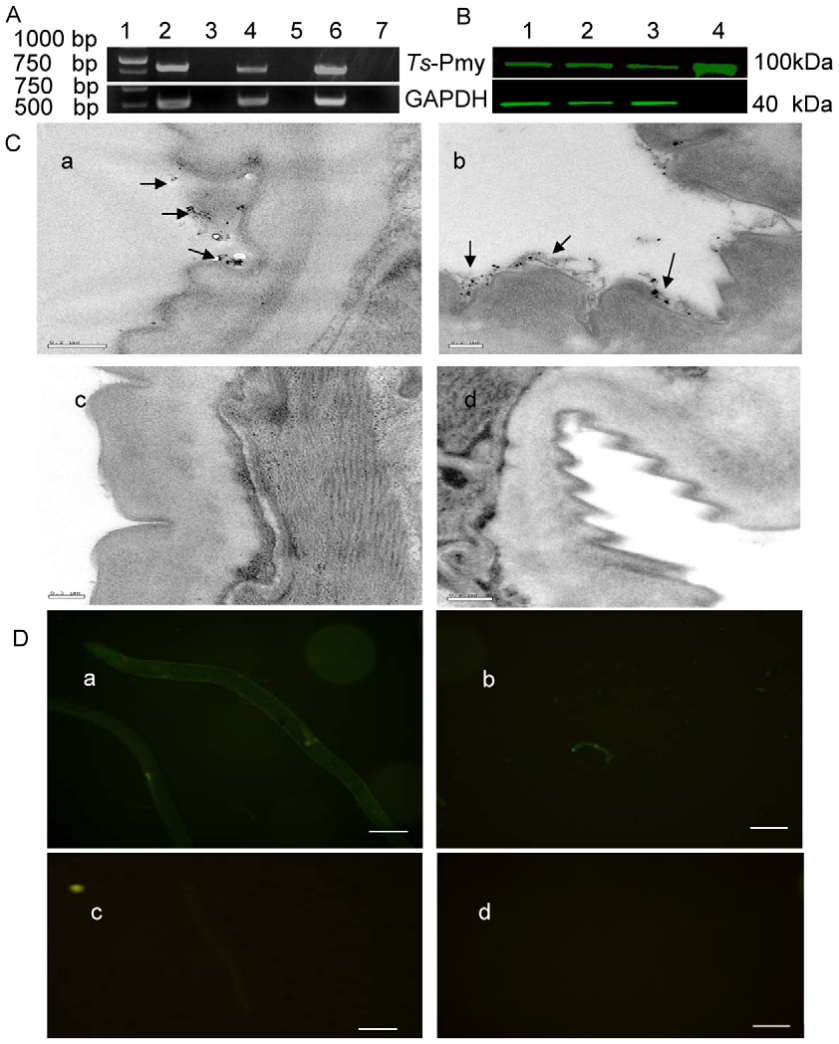
Expression and immunolocalization of *Ts*-Pmy in the different developmental stages of *T. spiralis*. (A) RT-PCR showing mRNA transcription of *Ts*-Pmy and GAPDH (control) from the different developmental stages of *T. spiralis*. Lane 1, DNA Marker; Lane 2, ML; Lane 4, adult worm; Lane 6, NBL. Lanes 3, 5 and 7: non-reverse transcriptase negative controls. (B) Western blot detection of native *Ts*-Pmy and GAPDH (control) expressed in different developmental stages of *T. spiralis* by specific antibodies with the Odyssey two color infrared imaging system. Lane 1, ML extracts (8 µg); Lane 2, adult worm extracts (8 µg); Lane 3, NBL extracts (8 µg); Lane 4, purified r*Ts*-Pmy (500 ng). (C). Electron micrographs of *T. spiralis* immunolabeled with anti-r*Ts-*Pmy mAb. NBL (a) and adult worm (b) were incubated with mouse anti-r*Ts-*Pmy mAb or normal mouse serum (c and d) and then with gold-conjugated protein A. Scale bars represent 0.2 µm. (D) Immunofluorescence micrographs of intact *T. spiralis* immunolabeled with rabbit antiserum against *Ts*-Pmy. Intact whole adult worms (a) and NBL (b) were incubated with anti-r*Ts-*Pmy rabbit serum or normal rabbit serum (c and d) and then with fluorescein isothiocyanate-conjugated goat anti-rabbit IgG. Scale bars represent 20 µm.

The transcription of *Ts*-Pmy mRNA at different developmental stages in *T. spriralis* was analyzed by RT-PCR with *Ts*-Pmy specific primers. *Ts*-Pmy mRNA was transcribed in all developmental stages (ML, NBL and adult worm; Figure 1A). The size of the amplified cDNA fragments was 900 bp, which is the same size as predicted by the DNA sequence. Western blot analysis showed that an approximately 100 kDa band was recognized by mAb 7E2 in the somatic extracts the three developmental stages of *T. spiralis* (ML, adult and NBL) (Figure 1B). The results show that *Ts*-Pmy is expressed in all three developmental stages (ML, adult worm and NBL) of *T. spiralis* at the level of both mRNA transcription and protein expression. Previously, immunofluorescent staining of worm sections demonstrated that *Ts*-Pmy was expressed on the surface of *T. spiralis* larvae [23]. Here, immunoelectron microscopy confirmed that *Ts*-Pmy was expressed on the outer membrane of the cuticle of the NBL (Figure 1Ca) and adult worm (Figure 1Cb). No significant staining was observed using normal mouse serum at the same dilution (c and d). Using immunofluorescence staining, *Ts*-Pmy was also observed on the surface of intact adult worms (Figure 1Da) and NBL (Figure 1Db).

### 2. Enhanced complement-mediated killing of NBL treated with anti- r*Ts*-Pmy rabbit serum

**Figure 2:**
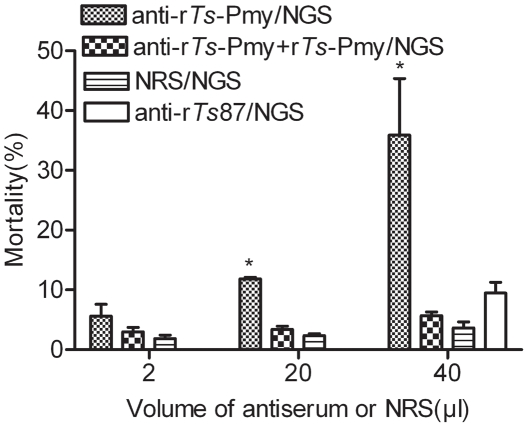
Enhanced complement-mediated killing of *T. spiralis* NBL incubated with anti-r*Ts-*Pmy rabbit serum. NBL were incubated with different amounts of anti-r*Ts-*Pmy rabbit serum, the same serum pre-absorbed with r*Ts*-Pmy or normal rabbit serum (NRS) in a total volume of 50 µL for 60 min before adding fresh normal guinea pig serum (NGS). In addition, 40 µL of anti-*Ts*87 rabbit serum was used as a control. Viability of NBL was observed under an inverted microscope. Values are expressed as the means ± SD for three independent experiments. *, statistically significant differences (p<0.05) between anti-r*Ts*-Pmy and anti-r*Ts*-Pmy + r*Ts*-Pmy or NGS control.

To determine whether *Ts*-Pmy expressed on the parasite surface protects NBL from being killed by the host complement, heat-inactivated rabbit anti-r*Ts*-Pmy serum was used to block *Ts*-Pmy on the NBL. The antiserum- or normal rabbit serum-treated NBL were challenged with the complement from fresh NGS. As shown in Figure 2, after being blocked with anti-r*Ts*-Pmy serum, NBL viability was significantly decreased following incubation with fresh NGS compared to those of the normal rabbit serum group. The increase in complement-mediated killing of NBL blocked with anti-r*Ts*-Pmy serum was observed in an antiserum dose-dependent manner. After being absorbed with r*Ts*-Pmy, the anti-r*Ts*-Pmy serum had a minimal or non-existent effect on the complement-mediated NBL killing. Incubating with rabbit antiserum against recombinant *Ts*87, a specific *T. spiralis* secreted protein [33], that is also located on the surface of worm [34], did not significantly increase complement-mediated NBL killing (Figure 2).

### 3. *In vivo* decrease in the infectivity of NBL treated with anti-r*Ts*-Pmy rabbit serum and complement

**Figure 3:**
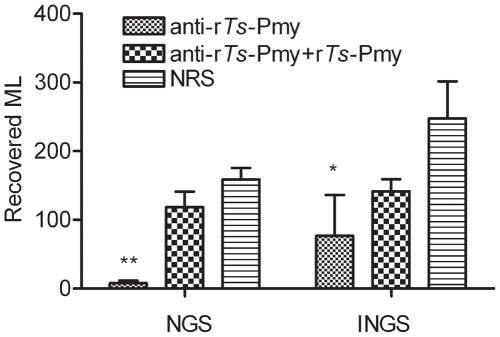
Recovered ML developed from NBL treated with anti-r*Ts*-Pmy rabbit serum and NGS. Two thousand NBL were incubated with anti-r*Ts*-Pmy rabbit serum, the same serum absorbed with r*Ts-*Pmy or NRS (100 µL), followed by NGS or INGS. Treated NBL were then injected into BALB/c mice. ML were recovered from the muscle tissues of mice 26 days after injection. Data represent the means ± SD from three independent experiments. **, indicate statistically significant differences (p<0.01) between the antiserum-treated/NGS group and protein absorbed serum/NGS or NRS/NGS group; * indicate statistically significant differences (p = 0.048) between the antiserum-treated/INGS group and NRS/INGS group (n = 8 in each group).

The number of ML recovered from mouse muscle was reduced by 95.1% for those NBL treated with anti-r*Ts*-Pmy rabbit serum and NGS complement compared with those treated with normal rabbit serum/NGS 26 days after being transferred intravenously into mice (Figure 3; *p = *0.006) . No significant reduction was observed for ML developed from NBL treated with r*Ts*-Pmy pre-absorbed anti-r*Ts*-Pmy serum. Heat-inactivated NGS (INGS) had a smaller killing effect on NBL treated with anti-r*Ts*-Pmy rabbit serum (72.0% ML reduction) or with the same serum pre-absorbed with r*Ts*-Pmy (48.5% ML reduction) compared with the normal rabbit serum control (Figure 3; *p = *0.048). These studies further indicated that *Ts*-Pmy on the surface of NBL may protect larvae from complement-mediated killing.

### 4. Inhibition of complement-mediated hemolysis by r*Ts*-Pmy

**Figure 4:**
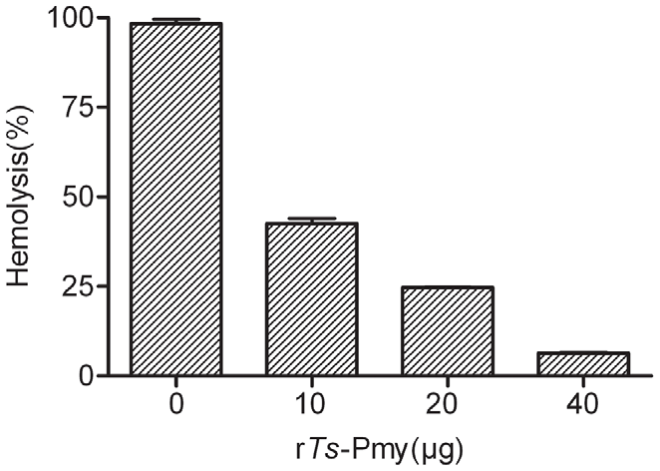
Inhibition of complement-mediated lysis of E_R_ by r*Ts*-Pmy. Various amounts of r*Ts*-Pmy (0, 10, 20 or 40 µg) were incubated with fresh NGS(6%), and then fresh E_R_ were added (3×10^8^ cells). The amount of hemoglobin released into the supernatant was measured at 412 nm, and the percent lysis was calculated. The results are shown as the means ± SD for three independent experiments.

Complement-mediated E_R_ lysis via the alternative pathway was significantly inhibited by adding r*Ts*-Pmy in a dose-dependent manner (*p*<0.001). E_R_ lysis was reduced by 55.7% when 10 µg of r*Ts*-Pmy was added and by 91.9% when 40 µg of r*Ts*-Pmy was added (Figure 4).

### 5. Binding of r*Ts*-Pmy to human C8 and C9

**Figure 5:**
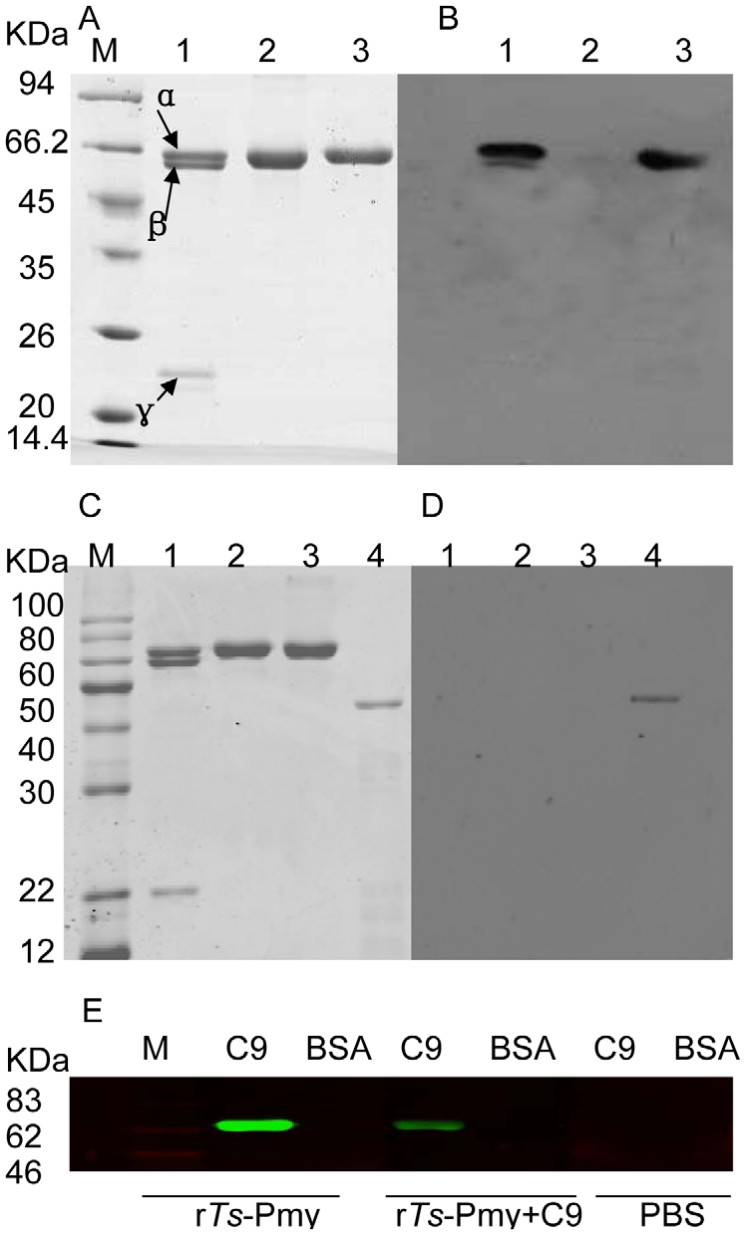
Determination of r*Ts*-Pmy binding to human C8 and C9. Human C8 (1 µg, Lane 1) and C9 (1 µg, Lane 3) and non-relevant BSA control (1 µg, Lane 2) were subjected to SDS-PAGE under reducing conditions and (A) stained with Coomassie blue, and then (B) transferred onto a PVDF membrane, incubated with r*Ts*-Pmy (5 µg/mL) and detected with anti-r*Ts-*Pmy mAb (50 ng/mL). The same materials that were added, plus non-relevant control protein r*Ts*87 (300 ng; Lane 4) were subjected to SDS-PAGE and (C) stained with Coomassie blue, and (D) transferred to an NC membrane, incubated with r*Ts*87 (5 µg/mL) and detected with anti-*Ts*87 rabbit serum (1∶4000). To determine the binding competition between soluble and immobilized C9 to r*Ts*-Pmy, r*Ts*-Pmy (5 µg/mL) was pre-incubated with C9 (30 µg/mL) for 1 hour before incubation with the membrane containing with C9 (1 µg) and BSA (1 µg), and was detected with anti-rTs-Pmy mAb (50 ng/mL) (E). M:standard protein marker.

**Figure 6:**
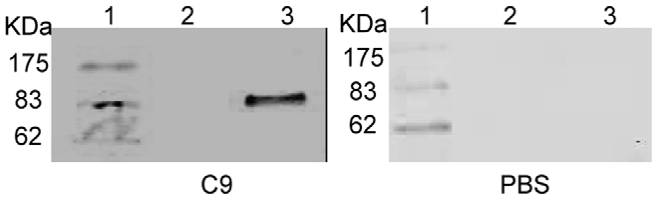
Binding of soluble C9 to immobilized r*Ts*-Pmy. BSA (Lane 2) and r*Ts*-Pmy (Lane 3) were transferred to a PVDF membrane, incubated with soluble C9 (left) or PBS only (right) and detected with monoclonal anti-C9 antibody.

After being transferred to a membrane, the human C8 and C9 were probed with r*Ts*-Pmy and detected with anti-r*Ts*-Pmy mAb. Western blot analysis demonstrated that r*Ts*-Pmy strongly bound to human C8 (α,β chains) and C9 (Figure 5B). The amount of r*Ts*-Pmy bound to the immobilized C9 was dramatically decreased (Figure 5E) after being absorbed with soluble C9. Reciprocally, the binding of C9 to r*Ts*-Pmy immobilized on a membrane was also demonstrated by incubating the membrane with soluble C9 and detecting with an anti-C9 mAb (Figure 6). In the control experiment, the same amount of recombinant *Ts*87 did not bind to C8 and C9 (Figure 5D).

### 6. Inhibition of C9 polymerization and poly-C9 formation on the E_R_ by r*Ts*-Pmy

**Figure 7:**
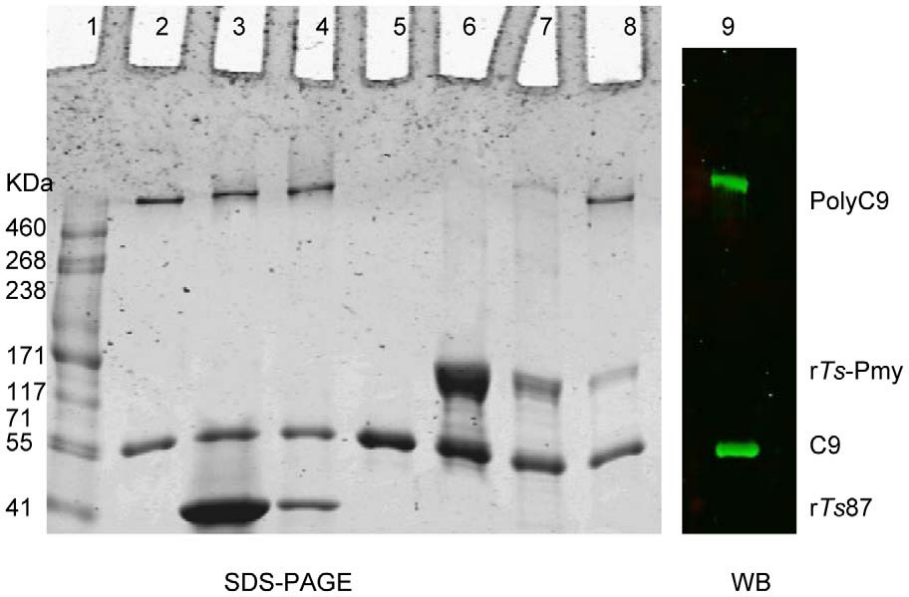
Inhibition of Zn^2+^-induced C9 polymerization by r*Ts*-Pmy. C9 (3 µg) was pre-mixed with various amounts of r*Ts*-Pmy (Lane 2, 0 µg; Lane 6, 10 µg; Lane 7, 2.5 µg and Lane 8, 1 µg) or r*Ts*87 (Lane 3, 10 µg; Lane 4, 2.5 µg) and incubated with 50 µM ZnCl_2_. Lane 5, blank C9 without adding Zn^2+^ as a negative control. Lane 1, standard protein marker. The reaction mixtures were analyzed by SDS-PAGE and Western blot with anti-C9 mAb for Lane 8 as shown in Lane 9.

**Figure 8:**
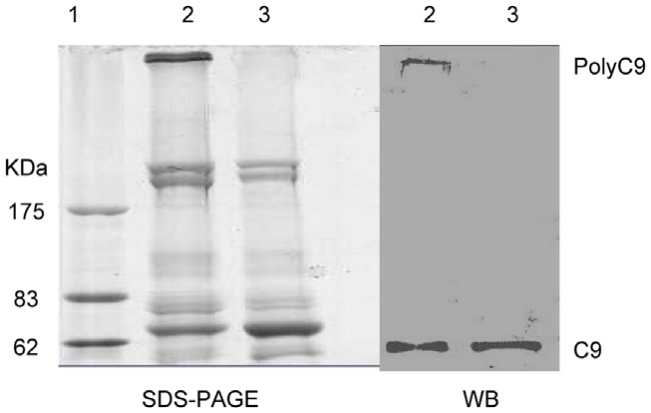
Inhibition of poly*-*C9 deposition on the surface of E_R_ by r*Ts*-Pmy. The NHS was incubated with r*Ts*-Pmy (Lane 3) or with buffer only (Lane 2) before adding fresh E_R_. Lane 1, standard protein marker. Membrane debris of lysed E_R_ was analyzed by SDS-PAGE under reducing conditions and Western blotting with anti-C9 mAb.

C9 polymerization leads to the creation of transmembrane channels that are critical for complement-mediated cytolysis [35]. To determine whether *Ts*-Pmy inhibits the Zn^2+^-induced C9 polymerization, C9 was mixed with various amounts of r*Ts*-Pmy and then incubated with 50 µM ZnCl_2_. As shown in Figure 7, r*Ts*-Pmy inhibited Zn^2+^-induced C9 polymerization in a dose-dependent manner. When the amount of r*Ts*-Pmy was increased to 10 µg, it completely inhibited the polymerization of 3 µg of C9 (Figure 7, lane 6). The same amount of recombinant *Ts*87 did not inhibit C9 polymerization. In addition, r*Ts*-Pmy inhibited the formation of the complement complex (poly-C9) on the surface of the E_R_ (Figure 8, lane 3).

## Discussion

Recent studies have shown that the complement system plays a role in granulocyte recruitment and parasite impairment in nematode infection prior to antibody production [36]. Complement components have been implicated in the killing of *Strongyloides stercoralis* larvae [37] and *Schistosoma mansoni* schistosomula [21]. The ability to evade complement attack is essential for the survival of tissue-dwelling nematodes within hosts. Many parasites, especially those living in or in contact with blood, seem to have developed parallel routes to escape complement attack [38]. *T. spiralis* infective larvae (ML), adults and NBL are able to bind to the complement components [14] and evade complement-mediated killing [15]. However, the mechanism by which *T. spiralis* evades complement-mediated killing is still not completely understood.

Paramyosin is an essential muscle protein in invertebrates, forming the core of thick myofilaments that determine the length and stability of muscles [17]. It has been suggested that the surface expression of paramyosin by *Schistosoma mansoni* [20] and *Taenia solium* [39] may inhibit the complement cascade of the immune system. Thus, in the present study, we examined the expression and localization of *Ts-*Pmy in the three developmental stages of *T. spiralis* and showed that *Ts*-Pmy is present on the outer membrane of the cuticle of the adults and NBL of *T. spiralis*. Our results were consistent with other observations that identified paramyosin in the tegument and on the surface of *Schistosoma mansoni, Schistosoma japonicum* [19], *Echinococcus granulosus* [40], *Taenia solium* [39] and *Fasciola hepatica* [41]. The existence of a surface-exposed form of paramyosin suggests a role as a potential modulator of the host immune system. It was previously observed that paramyosin on the surface of helminth parasites bound to the Fc of IgG [19] and IgA [42], collagen and at least three complement components, C1q [18], C8 and C9 [20]. The fact that *Ts*-Pmy is expressed on the outermost layer of the parasite indicates a possible role in the first line of defense against the host immune response.

Our data confirmed that surface-exposed *Ts*-Pmy binds to complement C8 and C9, which are important components of the complement activation cascade and comprise the membrane attack complex (MAC) [43]. Polymerization of C9 induced by Zn^2+^ was highly inhibited by r*Ts*-Pmy, indicating that the assembly of the MAC was impaired. The alternative complement pathway that activates the complement complex or poly-C9 on the rabbit erythrocytes (E_R_) was also greatly inhibited by r*Ts*-Pmy. These studies suggest that *Ts*-Pmy binding to C8 and C9 inhibits the assembly and formation of the MAC, creating an effective strategy for the parasite to evade complement attack [28]. Similar strategies are also adopted by *Schistosoma mansoni* [28], *Entamoeba hisolytica* [44], *Herpesvirus saimiri* [45] and *Trypanosoma cruzi* [46]. Functional analysis in this study revealed that r*Ts*-Pmy inhibited the lysis of E_R_ triggered by guinea pig serum, indicating that the binding of *Ts*-Pmy to C8/C9 or other complement elements inhibits the complement activation pathways. Whole *T. spiralis* worms also inhibited of complement-mediated hemolysis [14], suggesting that native *Ts*-Pmy or other modulating molecules on the surface of the worm might have similar functions as complement inhibitors. Such observations are consistent with the findings of other studies of paramyosin in *Schistosoma mansoni* [20], [21].

Additional evidences from this study show that native *Ts*-Pmy on the surface of *T. spiralis* effectively protected NBL from attack by host complements. Blocking *Ts*-Pmy on the surface of *T. spiralis* with anti-r*Ts*-Pmy antiserum reduce of NBL viability when under attack by complement *in vitro*. *In vivo* passive transfer experiments with NBL treated with anti-*Ts*-Pmy antiserum and NGS also showed a significant reduction in the number of NBL that developed into ML in mouse muscle, indicating that the infectivity of these larvae after *Ts*-Pmy blocked by antibodies was seriously impaired.

Our studies suggest that the outer membrane form of paramyosin expressed by *T. spiralis* has a role in host immunomodulation, presumably by inhibiting the formation of the MAC and thereby protecting the parasite from being damaged by activated complement. This modulation is an effective survival strategy for *T. spiralis* to live within its host. Disruptions of the immunomodulatory function of *Ts*-Pmy could be explored as an alternative strategy to control *T. spiralis* infection. As a result, *T. spiralis* paramyosin is under further evaluation as a potential laboratory reagent to study host complement function and as a potential vaccine antigen. The specific epitope in *Ts*-Pmy that reacts with the complement or elicits protective immunity is currently being defined.
